# Prediction of Positions of Active Compounds Makes It Possible To Increase Activity in Fragment-Based Drug Development

**DOI:** 10.3390/ph4050758

**Published:** 2011-05-20

**Authors:** Yoshifumi Fukunishi

**Affiliations:** 1 Biomedicinal Information Research Center (BIRC), National Institute of Advanced Industrial Science and Technology (AIST)/ 2-3-26, Aomi, Koto-ku, Tokyo 135-0064, Japan; E-Mail: y-fukunishi@aist.go.jp; Tel.: +81-3-3599-8290; Fax: +81-3-3599-8099; 2 Pharmaceutical Innovation Value Chain, BioGrid Center Kansai/ 1-4-2 Shinsenri-Higashimachi, Toyonaka, Osaka 560-0082, Japan

**Keywords:** FBDD, protein-compound docking, drug design, fragment growth, virtual screening, structure-based drug screening

## Abstract

We have developed a computational method that predicts the positions of active compounds, making it possible to increase activity as a fragment evolution strategy. We refer to the positions of these compounds as the active position. When an active fragment compound is found, the following lead generation process is performed, primarily to increase activity. In the current method, to predict the location of the active position, hydrogen atoms are replaced by small side chains, generating virtual compounds. These virtual compounds are docked to a target protein, and the docking scores (affinities) are examined. The hydrogen atom that gives the virtual compound with good affinity should correspond to the active position and it should be replaced to generate a lead compound. This method was found to work well, with the prediction of the active position being 2 times more efficient than random synthesis. In the current study, 15 examples of lead generation were examined. The probability of finding active positions among all hydrogen atoms was 26%, and the current method accurately predicted 60% of the active positions.

## 1. Introduction

In the drug-development process, after getting a set of seed compounds, the next step is lead generation. The major purpose of the lead generation is enhancement of the affinity of the seed compound to the target protein. In the lead generation process, the useless portion of the seed compound is reduced, while the necessary part is attached to the seed compound. In some cases, the scaffold is replaced by another scaffold. Usually, the QSAR method is applied to the seed and its derivatives to increase the affinity. The protein-compound complex structure is also analyzed in the lead generation.

Recently, fragment-based drug development (FBDD) has become popular. In FBDD, the most frequently used techniques are fragment linking, fragment evolution, fragment merging, and fragment growth [1-7]. In the fragment growth technique, which is the most popular method, additional fragments are attached to the seed compound by chemical modification. In the FBDD process, reducing the size of the seed compound is not the main tactic. Similar to conventional lead generation, the QSAR method is applied to the seed and its derivatives to increase the affinity, and the protein-compound complex structure is also analyzed in the lead generation.

In fragment evolution, one of the most important issues is predicting the position that can increase the affinity by chemical modification. In the lead generation process, the number of atoms is increased by 1.5 times from the seed compound to the lead compound, and the hydrophobicity is increased by the addition of hydrophobic groups to the seed compound [1]. If the protein-ligand complex structure is predicted by a docking study, the information can be helpful in designing the lead compound. However, prediction of the protein-ligand complex structure is difficult. Usually, the prediction accuracy of the cross-docking test by docking programs is approximately 20-30% [8, 9].

Especially in the case of the FBDD, a docking study of fragments is difficult, since the fragments are too small to obtain a stable protein-compound complex structure by docking study. Previously, we proposed the FSRG (fragment screening by replica generation) method, in which virtual side chains are attached to the fragments to enable the docking study [10]. In the current study, we developed a computational method that can predict position and increase the affinity by chemical modification for the fragment evolution method.

There have been many *de novo* design programs reported, such as LEGEND [11], LUDI [12], SPROUT [13], HOOK [14], GrowMol [15], PRO-LIGAND [16], CONCERT [17], LEA3D [18] and AutoGrow [19]. The differences between the current study and the previous studies are two points. The first point is that the previous studies reported the successful designed compounds for only one or two targets and the success rate was unclear. In the current study, the software was applied to the 15 targets and the success rate was evaluated. The second point is that the previous *de novo* studies overspecify the designed ligands. The designed compounds frequently meet the problem of synthetic accessibility. Some programs generate the compounds based on the known active compounds and these programs added chemical modification onto the given scaffolds. In some cases, medicinal chemists want to design ligands considering the availability of reagents and just want to know which position of compound must be modified. Thus, in the current study, the program was designed to suggest only the position of compound, which should be modified.

## 2. Method

Starting from an active fragment (seed compound) and the 3D structure of the target protein, we try to predict which atom should be modified chemically to increase the activity. In the current study, only hydrogen atoms and fluorine atoms were modified. While there are numerous varieties of chemical modifications, only a limited number of side chains (less than 80) were used in the current study for chemical modification. A set of virtual compounds were generated from the active fragment by artificial chemical modification, and the subsequent docking study was carried out to rank the virtual compounds according to the docking score. The modified position of the top-ranked virtual compound was predicted as the active position. The details of this method are described below.

**Figure 1:**
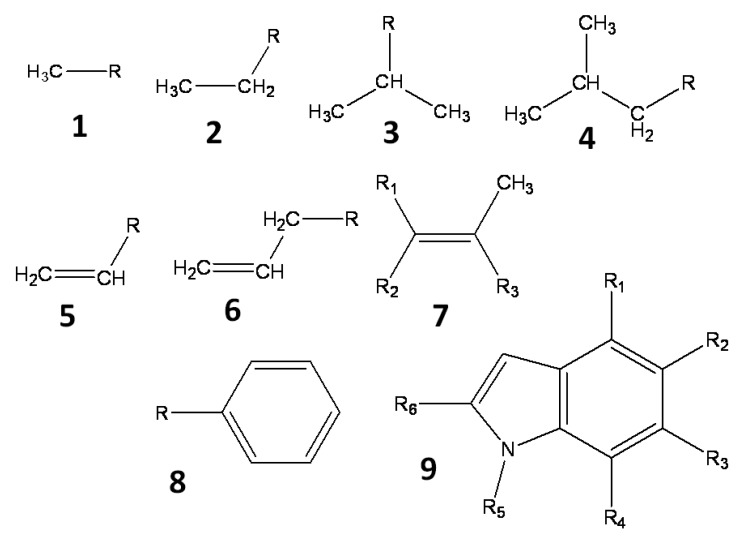
Side chain sets A, B, and C. All sets consist of the compounds 1-9 and their derivatives. **Set A**: For compound **1**-**9**, R or one of the R_i_s is directly attached to the active fragment and the other R_i_s are replaced by H. In addition, for compound **8**-**9**, R or one of the R_i_s is replaced by -CH_2_-R, -CH_2_-CH_2_-R, -CH=CH-R (R is the active fragment) and the other R_i_s are replaced by H. **Set B**: For compound **1**-**9**, R or one of the R_i_s is directly attached to the active fragment and the other R_i_s are replaced by H. In addition, for compound **8**-**9**, R or one of the R_i_s is replaced by -CH_2_-R, -CH_2_-CH_2_-R (R is the active fragment) and the other R_i_s are replaced by H. **Set C**: For compound **1**-**9**, R or one of the R_i_s is directly attached to the active fragment and the other R_i_s are replaced by H. In addition, for compound **8**-**9**, R or one of the R_i_s is replaced by -CH_2_-R (R is the active fragment) and the other R_i_s are replaced by H.

All hydrogen atoms and fluorine atoms of the active fragment were replaced by side chains, one by one. The side chains are small groups (methyl, ethyl, phenyl, *etc*) and their derivatives. Three sets of side chains (sets A, B and C) were prepared, and these sets, A, B, and C, consisted of 78, 38, and 25 side chains, respectively. These side chains, which are summarized in Figure 1, are small hydrocarbons including up to two aromatic rings, and they do not include heteroatoms. The side chains are prepared manually and arbitrary. These side chains were summarized in the supporting information.

These side chains are introduced into the active fragment by the BindMol program, which is an in-house program. If the attached side chain comes into contact with an atom of the seed compound (intra-molecular atomic conflict), such a compound is not generated. The atomic coordinates of the generated virtual compound are optimized by an energy minimization calculation in vacuum. The Cosgene/myPresto program is used for energy minimization with a general AMBER force field, and the dielectric constant is set to 4R, where R is the inter-atomic distance [20]. The atomic charges are calculated by the Gasteiger method [21, 22].

The protein-compound docking simulation is performed with the Sievgene/myPresto program [23]. Each generated virtual compound is docked to the target protein by the flexible docking method and the affinity of each virtual compound is evaluated by the docking score. The docking pocket of each protein was indicated by the coordinates of the original ligand. Hydrogen atoms were added to the coordinates by tplgene/myPresto. The atomic charges of the proteins were the same as those of AMBER parm99 [24]. For flexible docking, the Sievgene program generated up to 100 conformers for each compound, and a 120×120×120 grid is applied to the scoring grid. The atomic coordinates of the target protein were fixed. The protonated states of the proteins and compounds are the dominant ion forms at pH 7. Finally, the virtual compounds are sorted according to their docking scores. The modified position of the top ranked compound among the all virtual compounds is the predicted active position.

## 3. Results

### 3.1. Single target protein structure was used

**Figure 2:**
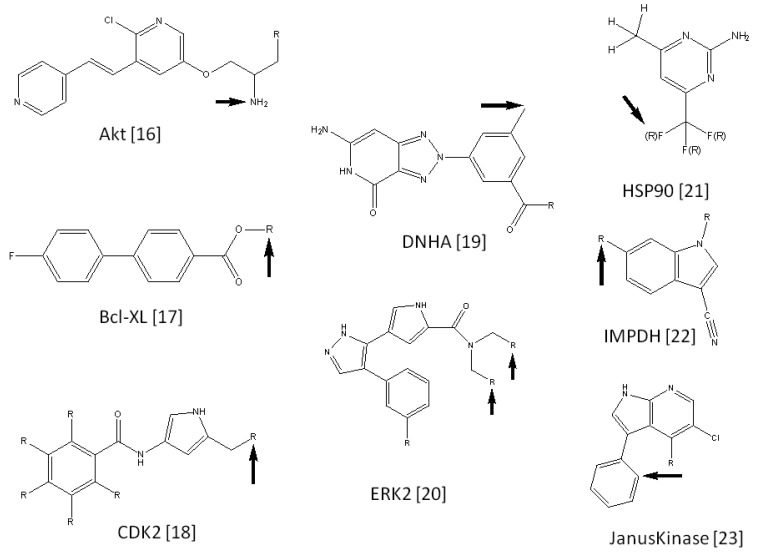

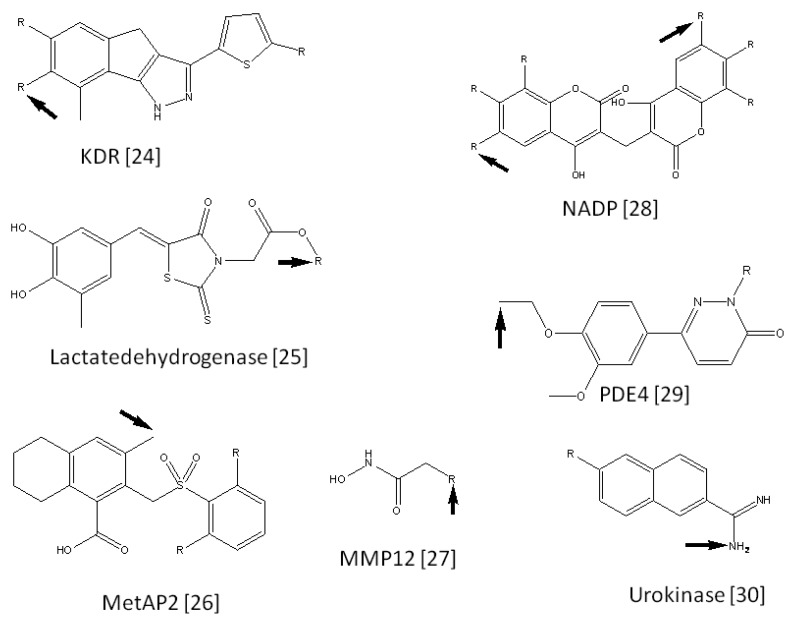
True active positions and predicted active positions. “R” represents the true active position, and the arrow represents the predicted active position by the current method by using side chain set A.

We collected 15 FBDD examples from literature reports [25-39]. Each example consisted of a seed compound, lead compounds derived from the seed compound, and 3D coordinates of the target protein. The seed compounds were suggested by the literature [1,40]. The current procedure was applied to these 15 target proteins. These target names are summarized in Table 1, along with the number of virtual compounds generated for each target. Figure 2 shows the seed compounds of these target proteins. The active positions of these compounds are also shown in Figure 2. Figure 2 also shows the predicted active positions by the current calculation. The probability of predicting accurate active positions is summarized in Table 2. On average, the probability of finding active positions among all hydrogen atoms was 26.32% by random prediction. On the other hand, the current method predicted 60.0% of the active positions when side chain set A was used. The prediction is approximately two times more efficient than a random selection of active positions. As far as the second top predicted position is considered in addition to the top ranked position, the probability of finding active positions among all hydrogen atoms was 45.71% by random prediction. On the other hand, the current method predicted 66.67%, 46.67% and 46.67% of the active positions when side chain sets A, B and C were used, respectively. These values were bigger than the probability by the random prediction, but the advantage of the current method is not significant anymore.

The prediction accuracy increased with increases the number of attached side chains. The prediction accuracy obtained with set A was better than that with sets B and C. Thus, the prediction accuracy should be improved by increasing the number of side chains or the variety of side chains.

The used virtual side chains (sets A, B and C) were not hydrophilic groups but hydrophobic groups that were hydrocarbons. In the fragment evolution process, hydrophobic groups are usually added to the active fragment compound to increase the activity [40]. It appears reasonable to use simple hydrophobic groups for chemical modification, while the variations of chemical modification are infinite.

### 3.2. Multiple target structures were used

In addition to the single target protein, multiple target protein structures were examined. These proteins were extracted from the PDB. The used protein structures are summarized in Table 2. Each protein was prepared for docking in the same manner described in the Methods section. The docking scores for all protein structures were merged and re-ranked based on the docking score. The results are summarized in Table 2. When side chain set A was used, the prediction accuracy was 66%, which is the same value obtained from the single target protein structure.

### 3.3. Ranking of true lead compounds

To estimate the limitations of the prediction accuracy, the true lead compounds were added to the virtual compounds. A single target protein structure was used. The compounds were docked to the target protein, and these compounds were ranked according to the docking score. If the docking scores are accurate, the true lead compounds should be ranked at the first positions. The results are summarized in Table 3. The true lead compounds appeared at the first rank with a probability of 60%, while this probability would be 2.8% by random selection. The docking study actually worked, but the prediction was not perfect. This 60% probability should be considered the upper limit of the current prediction method.

## 4. Discussion

On average, the probability of active positions among all hydrogen atoms was 26.32%, and the current method predicted 60.0% of the active positions. Considering that the accuracy of cross-docking by Sievgene is only 25%, the prediction accuracy of the current method is high. Our previous study shows that the virtual screening of fragment is difficult by docking study but that if a virtual side chain is added to the fragment compound, virtual screening of the modified fragment compound becomes easy [10]. As such, the accuracy is improved by the addition of a virtual side chain to fragment compounds. In the current study, the prediction accuracy would have been improved by the addition of virtual side chains to the active fragment compound.

The prediction accuracy obtained by side chain with set C was much better than that with sets A and B. The difference between the sets was that the side chains of set A included a C=C structure. This structure mimics that of amide or ester structures. Since the major contribution of the sievgene docking score is the ASA term and the electrostatic interaction is not as important, the size and shape of the group/compound is important in the sievgene docking score [10].

The prediction accuracy reached 60%, but no higher. Even if multiple structures were used, the prediction accuracy was not improved. In *in-silico* drug screening, the ensemble docking method has been used to consider features of protein flexibility such as induced-fitting. In an ensemble docking study, many protein structures are prepared for the docking study and each structure gives an *in-silico* drug screening result. We can obtain many screening results, but only a limited number of them can be reliable. How to select the reliable screening result from the many results is a serious problem. Ensemble docking studies have shown that the docking score does not consistently provide reliable results or true hit (active) compounds [41-44]. The same phenomenon should have occurred in the current study, and the docking scores were not good enough to predict the active positions.

Since comprehensive chemical modification is almost impossible, the reported active positions should correspond to one of the registered active positions. The same as the probability of true active positions, the accuracy of prediction should be underestimated. That is, even though a true active position is predicted, if the position is not reported (the position is not included in the registered active positions), the prediction is judged to be a failure. These chemical modifications are restricted by the synthetic accessibility, and the analysis of this current study is somewhat ambiguous.

## 5. Conclusions

We have developed a computational method that predicts the positions of seed compounds that should be chemically modified in the fragment evolution method. In the current method, to predict the active position, all hydrogen atoms are replaced by small side chains. Three sets of side chains were prepared manually. These virtual compounds were docked to a target protein, and the docking scores (affinities) were examined. The hydrogen atom that gave the virtual compound with good affinity was determined to be the active position that should be replaced to generate a lead compound. This method worked well. The prediction of active position was two times more efficient than random synthesis. In the current study, 15 examples of lead generation were examined. The probability of active positions among all hydrogen atoms was 26%, and the current method predicted 60% of the active positions.

## Figures and Tables

**Table 1:** Active position prediction accuracy by the current method.

**Target protein**	**PDB ID**	**Active fragment**		**Set A**		**Set B**		**Set C**	
		**No. of H/F^a^**	**No. of active sites^b^**	**No. of compds^c^**	**Rank^d^**	**No. of compds**	**Rank**	**No. of compds**	**Rank**
Akt	2UZT	17	3	1311	2	647	2	425	2
Bcl-XL	1YS1	10	1	726	1	373	2	245	2
CDK2	1VYZ	12	8	859	1	419	1	274	1
DNHA	2NM2	8	1	618	63	302	12	196	12
ERK2	2OJG	17	8	1159	1	583	1	386	1
HSP90	1BYQ	10	3	695	1	344	3	225	3
IMPDH	1NF7	7	3	469	1	230	1	150	1
Janus kinase	3JY9	10	1	777	28	380	17	248	17
KDR	1T46	10	3	757	1	379	1	249	1
Lactate dehydrogenase	1ARZ	10	1	701	1	344	9	225	9
MetAP2	1YW7	16	2	1273	36	627	10	407	10
MMP12	1Y93	6	3	392	1	192	1	125	1
NADP	2F10	13	6	839	1	404	3	256	3
PDE4	1MKD	14	1	1094	134	534	33	350	33
Urokinase	1ETF	11	1	727	43	338	31	209	28
Average			26.32%		60.00%		33.33%		33.33%

a number of H/F atoms of active fragment;

b number of true active positions of active fragment;

c number of generated virtual compounds;

d rank of the virtual compound that precisely predict the true active position

**Table 2:** Prediction results with multiple target protein structures.

**Target**	**PDB ID**					**Rank^a^**
Bcl-XL	1YS1	1YSG	1YSN	1YSW	2YXJ	1
ERK2	2OJG	2OJI	2OJJ	2OK1		1
LFA-1	1XDD	1XDG				1
MetAP2	1YW7	1YW8				58
NADP	2F10	3JSX				1
PDE4	1MKD	1Q9M				28
	Average					66.67%

a rank of the virtual compound that precisely predicts the true active position

**Table 3:** Rank of the true lead compounds.

**Target protein**	**PDB ID**	**No. of compoundsa**	**No. of true leads**	**Rankb**
Akt	2UZT	1361	50	41
Bcl-XL	1YS1	727	1	1
CDK2	1VYZ	900	41	1
DNHA	2NM2	634	16	1
ERK2	2OJG	1192	33	334
HSP90	1BYQ	699	4	1
IMPDH	1NF7	497	28	1
JanusKinase	3JY9	778	1	343
KDR	1T46	805	48	1
Lactatedehydrogenase	1ARZ	704	3	1
MetAP2	1YW7	1381	108	1
MMP12	1Y93	393	1	8
NADP	2F10	867	28	298
PDE4	1MKD	1102	8	303
Urokinase	1ETF	754	27	1
Average			2.80%	60.00%

a number of generated virtual compounds;

b rank of the virtual compound that precisely predicts the true active position

## Supplementary Files

Supplementary File 1 PDF-Document (PDF, 25 KB)
