# Supplementary material for: Prediction of Positions of Active Compounds Makes It Possible To Increase Activity in Fragment-Based Drug Development
Source: Pharmaceuticals (Basel). 2011 May 20;4(5):758–69. doi: 10.3390/ph4050758 (PMC4055877; doi:10.3390/ph4050758)
Supplement: Supplementary File 1 — PDF-Document (PDF, 25 KB) [file pharmaceuticals-04-00758-s001.pdf]

Supplementary Materials

```
#
# side chain set A
#
[Xx]c1ccccc1
[Xx]Cc1ccccc1
C([Xx])Cc1ccccc1
[Xx]c1nc2ccccc2n1
[Xx]c1cccc2ncnc12
[Xx]c1ccc2ncnc2c1
c1ccc2c(c1)ncn2[Xx]
c1cc([Xx])c2ncnc2c1
c1cc2ncnc2cc1[Xx]
c12ccccc1nc(n2)CC[Xx]
c12ccccc1n(cn2)CC[Xx]
c12ncnc1c(ccc2)CC[Xx]
c12ncnc1cc(cc2)CC[Xx]
c12ncnc1ccc(c2)CC[Xx]
c12ncnc1cccc2CC[Xx]
[Xx]Cc1nc2ccccc2n1
[Xx]Cc1cccc2ncnc12
[Xx]Cc1ccc2ncnc2c1
c12ncnc1cc(cc2)C[Xx]
c12ncnc1c(C[Xx])ccc2
c12ccccc1n(cn2)C[Xx]
c1([Xx])nc2ccccc2n1C=C
c1nc2c([Xx])cccc2n1C=C
c1nc2cc([Xx])ccc2n1C=C
c1nc2ccc([Xx])cc2n1C=C
c1nc2cccc([Xx])c2n1C=C
c1nc2ccccc2n1C([Xx])=C
c1nc2ccccc2n1C=C[Xx]
c1nc2ccccc2n1C=C[Xx]
c1([Xx])nc2cccc(C=C)c2n1
c1nc2cccc(C=C)c2n1[Xx]
c1nc2c([Xx])ccc(C=C)c2n1
c1nc2cc([Xx])cc(C=C)c2n1
c1nc2ccc([Xx])c(C=C)c2n1
c1nc2cccc(C(=C)[Xx])c2n1
c1nc2cccc(C=C[Xx])c2n1
c1nc2cccc(C=C[Xx])c2n1
c1([Xx])nc2cccc(C=C)c2n1
c1nc2cccc(C=C)c2n1[Xx]
c1nc2c([Xx])ccc(C=C)c2n1
c1nc2cc([Xx])cc(C=C)c2n1
c1nc2ccc([Xx])c(C=C)c2n1
c1nc2cccc(C(=C)[Xx])c2n1
c1nc2cccc(C=C[Xx])c2n1
c1nc2cccc(C=C[Xx])c2n1
c1([Xx])nc2ccc(C=C)cc2n1
c1nc2ccc(C=C)cc2n1[Xx]
```

c1nc2cc(C=C)cc([Xx])c2n1
c1nc2cc([Xx])c(C=C)cc2n1
c1nc2ccc(C=C)c([Xx])c2n1
c1nc2ccc(cc2n1)C([Xx])=C
c1nc2ccc(C=C[Xx])cc2n1
c1nc2ccc(C=C[Xx])cc2n1
c1([Xx])nc2ccc(C=C)cc2n1
c1nc2ccc(C=C)cc2n1[Xx]
c1nc2cc(C=C)cc([Xx])c2n1
c1nc2cc([Xx])c(C=C)cc2n1
c1nc2ccc(C=C)c([Xx])c2n1
c1nc2ccc(cc2n1)C(=C)[Xx]
c1nc2ccc(C=C[Xx])cc2n1
c1nc2ccc(C=C[Xx])cc2n1
C([Xx])=Cc1ccccc1
C([Xx])=Cc1ccccc1
C=C([Xx])c1ccccc1
C=Cc1ccccc1[Xx]
C=Cc1cccc([Xx])c1
C=Cc1ccc([Xx])cc1
C=Cc1cccc([Xx])c1
C=Cc1ccccc1[Xx]
[Xx]C
C(C)[Xx]
CC([Xx])C
CCC[Xx]
C(=C)[Xx]
C([Xx])=CC
C([Xx])=CC
C=C([Xx])C
C=CC[Xx]
#
side chain set B
#
[Xx]c1ccccc1
[Xx]Cc1ccccc1
C([Xx])Cc1ccccc1
[Xx]c1nc2ccccc2n1
[Xx]c1cccc2ncnc12
[Xx]c1ccc2ncnc2c1
c1ccc2c(c1)ncn2[Xx]
c1cc([Xx])c2ncnc2c1
c1cc2ncnc2cc1[Xx]
c12ccccc1nc(n2)CC[Xx]
c12ccccc1n(cn2)CC[Xx]
c12ncnc1c(ccc2)CC[Xx]
c12ncnc1cc(cc2)CC[Xx]
c12ncnc1ccc(c2)CC[Xx]
c12ncnc1cccc2CC[Xx]
[Xx]Cc1nc2ccccc2n1
[Xx]Cc1cccc2ncnc12

[Xx]Cc1ccc2ncnc2c1
c12ncnc1cc(cc2)C[Xx]
c12ncnc1c(C[Xx])ccc2
c12ccccc1n(cn2)C[Xx]
C([Xx])=Cc1ccccc1
C([Xx])=Cc1ccccc1
C=C([Xx])c1ccccc1
C=Cc1ccccc1[Xx]
C=Cc1cccc([Xx])c1
C=Cc1ccc([Xx])cc1
C=Cc1cccc([Xx])c1
C=Cc1ccccc1[Xx]
[Xx]C
C(C)[Xx]
CC([Xx])C
CCC[Xx]
C(=C)[Xx]
C([Xx])=CC
C([Xx])=CC
C=C([Xx])C
C=CC[Xx]
#
side chain set C
#
[Xx]c1ccccc1
[Xx]Cc1ccccc1
C([Xx])Cc1ccccc1
[Xx]c1nc2ccccc2n1
[Xx]c1cccc2ncnc12
[Xx]c1ccc2ncnc2c1
c1ccc2c(c1)ncn2[Xx]
c1cc([Xx])c2ncnc2c1
c1cc2ncnc2cc1[Xx]
c12ccccc1nc(n2)CC[Xx]
c12ccccc1n(cn2)CC[Xx]
c12ncnc1c(ccc2)CC[Xx]
c12ncnc1cc(cc2)CC[Xx]
c12ncnc1ccc(c2)CC[Xx]
c12ncnc1cccc2CC[Xx]
[Xx]Cc1nc2ccccc2n1
[Xx]Cc1cccc2ncnc12
[Xx]Cc1ccc2ncnc2c1
c12ncnc1cc(cc2)C[Xx]
c12ncnc1c(C[Xx])ccc2
c12ccccc1n(cn2)C[Xx]
[Xx]C
C(C)[Xx]
CC([Xx])C
CCC[Xx]
